# RIZ1 is potential CML tumor suppressor that is down-regulated during disease progression

**DOI:** 10.1186/1756-8722-2-28

**Published:** 2009-07-14

**Authors:** Ashakumary Lakshmikuttyamma, Naoto Takahashi, Elodie Pastural, Emina Torlakovic, Hesham M Amin, Guillermo Garcia-Manero, Michael Voralia, Magdalena Czader, John F DeCoteau, C Ronald Geyer

**Affiliations:** 1 Cancer Stem Cell Research Group, University of Saskatchewan, Saskatoon, SK, Canada; 2 Department of Pathology, University of Saskatchewan, Saskatoon, SK, Canada; 3 Department of Biochemistry, University of Saskatchewan, Saskatoon, SK, Canada; 4 Department of Hematopathology, MD Anderson Cancer Center, University of Texas, Houston TX, USA; 5 Department of Leukemia, MD Anderson Cancer Center, University of Texas, Houston TX, USA; 6 Department of Oncology and Hematology, Saskatchewan Cancer Agency Saskatoon, SK, Canada; 7 Stem Cell Transplant Program, Saskatchewan Cancer Agency Saskatoon, SK, Canada; 8 Department of Pathology and Laboratory Medicine, Indianapolis, IN, USA

## Abstract

**Background:**

RIZ1 expression and activity are reduced in many cancers. In AML cell lines and patient material, RIZ1 expression is reduced relative to normal bone marrow. In chronic myelogenous leukemia (CML), blastic transformation is associated with loss of heterozygosity in the region where RIZ1 is located. RIZ1 is a PR domain methyltransferase that methylates histone H3 lysine 9, a modification important for transcriptional repression. In CML blast crisis cell lines RIZ1 represses insulin-like growth factor-1 expression and autocrine signaling. Together these observations suggest that RIZ1 may have a role in the chronic phase to blast crisis transition in CML.

**Results:**

In CML patient material, we observed that RIZ1 expression was decreased during progression from chronic phase to blast crisis. RIZ1 was expressed in mature myeloid and CD34^+ ^cells demonstrating that decreased RIZ1 expression in blast crisis is not due to an increased immature cell population. Expression of RIZ1 CML blast crisis cell lines decreased proliferation, increased apoptosis, and enhanced differentiation.

**Conclusion:**

RIZ1 is a candidate tumor suppressor gene whose expression is decreased in blast crisis. Loss of RIZ1 activity results in decreased apoptosis and differentiation and enhanced proliferation. Together these results suggest that loss of RIZ1 expression will lead to an increase in myeloid blast cell population resulting in CML progression.

## Background

Molecular mechanisms responsible for driving the transition of chronic myelogenous leukemia (CML) from chronic phase to blast crisis are not well characterized. CML evolves from a chronic phase that is associated with the Philadelphia chromosome to a blast crisis phase, which is associated with additional chromosome or molecular aberrations. Evolution to blast crisis is correlated with frequent loss of heterozygosity at chromosome region 1p36 [1]. *RIZ1*, a PR domain methyltransferase, is located at 1p36. RIZ1 methylates histone H3 lysine 9, a modification important for transcriptional repression [2]. RIZ1 expression and activity are reduced in many human cancers by genetic and epigenetic mechanisms [3,4]. RIZ1 expression is reduced in acute myeloid leukemia [5] and the *RIZ1 *knockout mouse has a high incidence of diffuse large B-cell lymphoma [4]. RIZ1 also regulates IGF-1 signaling in CML blast crisis cell lines [6]. Together these data suggest that decreased RIZ1 expression may contribute to CML progression. We investigated whether RIZ1 expression was reduced during CML progression and whether RIZ1 induced phenotypes that support its role as a candidate tumor suppressor.

## Results and discussion

**Figure 1:**
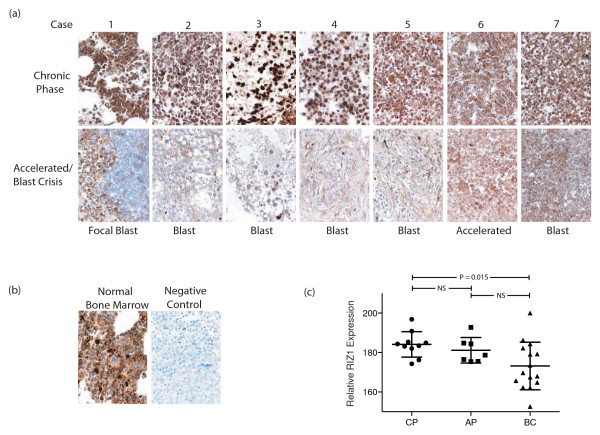
**RIZ1 expression in bone marrow of CML patients**. (**a**) Immunohistochemical analysis of matched bone marrow trephine biopsies and bone marrow aspirate clot samples from patients in chronic phase and accelerated phase or myeloid blast crisis using an anti-RIZ1 antibody. (**b**) RIZ1 expression in normal bone marrow and normal bone marrow staining in the absence of RIZ1 primary antibody (Negative control). (**c**) Immunohistochemical analysis of RIZ1 expression in unmatched patient bone marrow biopsies and clot sections from chronic phase (CP) (N = 10), accelerated phase (AP) (N = 7) and blast crisis (BC) (N = 15) using an anti-RIZ1 monoclonal antibody. Relative RIZ1 expression represents 3,3-diaminobenzidine chromagen intensity. Mean RIZ1 expression for each group is shown as a black line and errors bars represent the standard deviation.

We characterized RIZ1 expression in matched bone marrow biopsies from seven CML patients in chronic phase, accelerated phase, or myeloid blast crisis by immunohistochemistry (Fig 1a). Anti-RIZ1 antibody is specific for the N-terminus of RIZ1 and thus does not recognize the RIZ2 isoform [6]. Previously this antibody has been used to specifically detect RIZ1 in flow cytometry [6], Western analysis [6], and chromatin immunoprecipitation assays [2,6]. We observed strong cytoplasmic and nuclear RIZ1 expression during chronic phase in all cases, which was similar to RIZ1 expression in normal bone marrow (Fig 1a, b). Five of six cases in blast crisis had markedly reduced RIZ1 expression (Cases 1–5). In Case 1, the patient had focal blast crisis and RIZ1 expression was stronger in areas not involved in the blast foci. One blast crisis patient (Case 7) and an accelerated phase patient (Case 6) showed no appreciable change in RIZ1 expression. To validate these results further, we analyzed RIZ1 expression in a CML tissue microarray containing a larger cohort of unmatched bone marrow biopsies in chronic phase, accelerated phase, and blast crisis by immunohistochemistry. We observed a significant decrease in RIZ1 expression (P = 0.015) in blast crisis compared to chronic phase biopsies (Fig 1c). We did not observe any significant differences in RIZ1 expression between chronic phase and accelerated phase or between accelerated phase and blast crisis. The mean value for RIZ1 expression in blast crisis separates high and low RIZ1 expressing biopsies and was approximately equal to the lower standard deviation for RIZ1 expression in chronic phase. This is consistent with other molecular defects in the high RIZ1 expression biopsies contributing to the chronic phase to blast crisis transition. Abnormalities of proto-oncogenes, such as RAS and MYC, or of tumor suppressor genes, such as mutations of the *p53 *gene, absence of RB protein, and homozygous deletions of the *p16*^*INK4a *^gene, have been reported to occur during the chronic phase to blast crisis transition [7].

**Figure 2:**
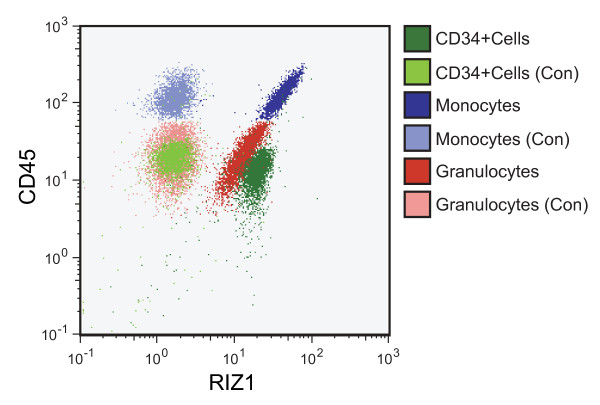
**RIZ1 expression in G-CSF mobilized peripheral blood**. Flow cytometry analysis of RIZ1 protein expression in granulocytes, monocytes, and CD34^+ ^cells. (Con) represents flow cytometry analysis in the absence of the RIZ1 primary antibody.

To confirm that low RIZ1 expression was correlated with myeloid blast crisis and not due to low RIZ1 expression in immature hematopoietic cells, we compared RIZ1 expression in immature and mature hematopoietic cells. We observed RIZ1 expression in both immature and differentiated cells in chronic phase and control bone marrow (Fig 1a). RIZ1 expression was maintained in the immature cells of two CML patients, one in accelerated phase with 15% blasts (Case 6) and the other in blast crisis (Case 7), indicating that low RIZ1 expression was not an inherent property of immature hematopoietic cells. We also measured RIZ1 expression in CD34^+ ^cells, granulocytes, and monocytes from G-CSF mobilized peripheral blood (Fig 2). RIZ1 was expressed in mature myeloid and CD34^+ ^cells, indicating that differences in RIZ1 expression in chronic phase and blast crisis were not a reflection of increased immature cell population in blast crisis.

The mechanism for decreased RIZ1 expression in CML blast crisis is not known. One possible explanation is that the RIZ1 promoter CpG island is aberrantly hypermethylated. In the CML blast crisis cell line, K562, the RIZ1 promoter is hypermethylated and addition of a methylation inhibitor, 5-aza-2'-deoxycytidine, induces RIZ1 expression [8]. Epigenetic silencing has also been reported to reduce RIZ1 expression in other cancers [3].

**Figure 3:**
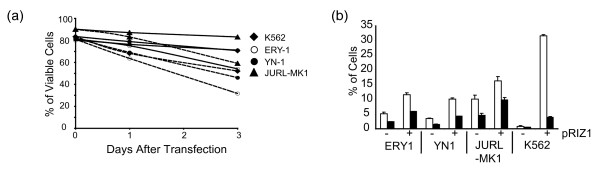
**Effect of RIZ1 expression on cell viability and apoptosis in CML myeloid blast crisis model cell lines**. (**a**) Viability assay for cell lines transfected with pRIZ1 (dashed line) or pcDNA3 control plasmid (solid line). (**b**) Annexin V assay of ERY-1, YN-1, JURL-MK1, and K562 one day after transfection with pRIZ1 (+) or pcDNA3 control plasmid (-). Percentages of apoptotic cells were detected using annexin V-FITC and PI staining. Total percentage of cells undergoing early and end stage apoptosis are indicated. White histogram represents cells in early apoptosis (FITC^+^, PI^-^). Black histogram represents cells that are in the end stage of apoptosis or that are already dead (FITC^+^, PI^+^). Error bars represent standard deviation from three independent experiments.

We used CML blast crisis cell lines, K562, YN-1, and ERY-1, which express immature erythroid cell features, and JURL-MK1, which can undergo megakaryocytic differentiation, as model systems analyzing the effects of RIZ1 expression. We previously used these cells to transiently express RIZ1 [6]. We monitored viability and apoptosis of RIZ1-transfected cell lines using trypan blue exclusion and annexin V assays, respectively. K562, YN-1, and ERY-1 were less viable when transfected with pRIZ1 than JURL-MK1 (Fig 3a). Transient transfection of pRIZ1 increased the number of cells undergoing early and late apoptosis in all cell lines (Fig 3b). Similar results have been reported for the forced expression of RIZ1 in breast cancer [9], hepatoma [10], and promyelocytic leukemia [11] cell lines, where RIZ1 expression causes cell cycle arrest and cell death and a decrease in proliferation.

**Figure 4:**
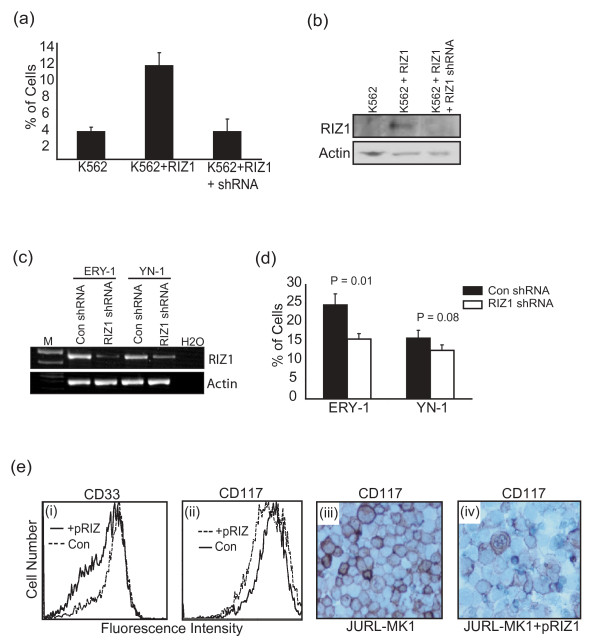
**Effect of RIZ1 expression on differentiation in CML myeloid blast crisis model cell lines**. (**a**) Benzidine staining assays comparing erythroid differentiation in K562 cells transfected with shRNA non-silencing control plasmid (K562), K562+RIZ1 cells transfected with shRNA non-silencing control plasmid (K562+RIZ1), and K562+RIZ1 cells transfected with pRIZ1shRNA (K562+RIZ1+shRNA). (**b**) Western analysis of RIZ1 expression in K562 transfected with shRNA non-silencing control plasmid (K562), K562+RIZ1 cells transfected with shRNA non-silencing control plasmid (K562+RIZ1), and K562+RIZ1 cells transfected with pRIZ1shRNA (K562+RIZ1+shRNA). (**c**) RT-PCR analysis of RIZ1 mRNA expression in ERY-1 and YN-1 transfected with shRNA non-silencing control plasmid (Con shRNA) or with pRIZ1shRNA (RIZ1 shRNA). Total RNA was reverse transcribed and cDNA amplified with RIZ1 and β-actin-specific primers. M represent DNA ladder and H2O represents RT-PCR reaction without template DNA. (**d**) Erythroid differentiation assay comparing ERY-1 and YN-1 after transfection with pRIZ1shRNA or shRNA non-silencing control plasmid (Con). Cell lines were transfected with pRIZ1shRNA or shRNA non-silencing control plasmid and cultured for three days. Histograms show the percentage of benzidine-positive cells that were scored by light microscopy. Error bars represent the standard deviation from three independent experiments. (**e**) CD33 and CD117 expression in JURL-MK1 cells as compared with JURL-MK1 cells expressing RIZ1 (JURL-MK1+pRIZ). JURL-MK1 was transfected with pRIZ1 or pcDNA3 control plasmid (con) and cultured for three days. Panel (**i**) shows the fluorescence intensity of phycoerythrin (PE)-conjugated antibody against CD33. Panel (**ii**) shows the fluorescence intensity of (PE)-conjugated antibody against CD117. Panels (**iii**) and (**iv**) show immunocytochemical staining using an anti-CD117 antibody in JURL-MK and JURL-MK1+pRIZ1 cells, respectively.

K562, YN-1, and ERY-1 express low levels of hemoglobin, reflecting their myeloid/erythroid progenitor phenotype. We used benzidine staining to monitor whether RIZ1 expression promotes erythroid differentiation. Transient expression of RIZ1 in K562, YN-1, and ERY-1 was too toxic to measure erythroid differentiation as the benzidine assay requires incubation times longer than one day. Previously, we generated a stable RIZ1 expressing K562 cell line (K562+RIZ1) that expresses less toxic levels of RIZ1 [6]. Stable expression of RIZ1 in K562 increases erythroid differentiation compared to K562 alone (Fig 4a). To confirm that RIZ1 is responsible for enhanced erythroid differentiation in K562+RIZ1 cell line, we measured erythroid differentiation in K562+RIZ1 transfected with a plasmid that expresses RIZ1 shRNA (pRIZ1shRNA). Expression of pRIZ1shRNA in K562+RIZ1 reduced RIZ1 protein expression [6] and erythroid differentiation to levels similar to K562 (Fig 4b). ERY-1 and YN-1 have higher endogenous RIZ1 expression than K562 and therefore we monitored the effect of pRIZ1shRNA on erythroid differentiation directly in these cell lines. Expression of pRIZ1shRNA in ERY-1 and YN-1 decreased RIZ1 expression and erythroid differentiation (Fig 4c, d).

We analyzed the effect of RIZ1 expression on megakaryocytic differentiation in JURL-MK1 cells by measuring changes in CD33 and CD117 using flow cytometry and immunocytochemistry. CD33 and CD117 are present in myeloid progenitors and their expression decreases with maturation and differentiation. Transient transfection of pRIZ1 into JURL-MK1 decreased CD33 and CD117 expression as monitored by flow cytometry (Fig 4e). Immunohistochemical staining using CD117 antibody also shows that transient transfection of pRIZ1 into JURL-MK1 decreased CD117 expression (Fig 4e).

## Conclusion

These results build upon previous observations that a putative CML tumor suppressor gene is present at 1p36 that exhibits loss of heterozygosity during transformation from chronic phase to blast crisis [1]. We propose a model whereby in chronic phase CML there is an expansion of BCR/ABL positive CML progenitor cells that maintain the ability to undergo apoptosis and differentiation. Epigenetic or genetic aberrations in RIZ1 expression and activity result in a blockage of apoptotic and differentiation pathways, which causes expansion of the myeloid blast cell population.

## Methods

### Cell Lines, CD34^+ ^Cells, and CML Patient Material

K562 is from ATCC (Manassas, VA, USA), JURL-MK1 is from DSMZ (Braunschweig, Germany), YN-1, ERY-1, and K562+RIZ1 have been described previously [6]. CD34^+ ^cells were purified from G-CSF mobilized peripheral blood using an AutoMACs Separator with a Direct CD34 Progenitor Cell Isolation Kit from Miltenyi Biotech (Auburn, CA, USA). Fixed bone marrow specimens from CML chronic phase patients that progressed to accelerated phase or blast crisis were obtained from the Department of Pathology and Laboratory Medicine (Indiana University). Patients were diagnosed in chronic phase between 1997–2000 and in accelerated phase or blast crisis between 2000–2004. Unmatched patient CML bone marrow biopsies and clot sections were obtained from the MD Anderson Cancer Center as described previously [12]. Patient samples were obtained with informed consent according to institutional review board guidelines.

### Cell Line Transfections and Assays

Plasmids were transfected into cell lines using the Nucleofector system (Amaxa, Gaithersburg, MD, USA). Transfection efficiencies for CML cell lines were: K562 – 74.5% ERY-1 – 68.6%, YN-1 – 75.3, JURL-MK1 – 77%. pRIZ1 (p3RIZRH4.1) was from Steele-Perkins *et al*, [4] and pCDNA3 was from Invitrogen (Carlsbad, CA, USA). pRIZ1shRNA and shRNA non-silencing control vector were from OPEN Biosystems (Huntsville, AL, USA). Cell viability, apoptosis, and hemoglobin staining were assayed using Trypan blue dye exclusion, Annexin V-FITC Apoptosis Detection Kit (BD Biosciences, San Jose, CA, USA), and benzidine staining, respectively.

### Flow Cytometry

Conjugated antibodies used for surface analysis of CD45, CD34, CD33, and CD117 expression are from Beckman Coulter (Fullerton, CA, USA). Intracellular RIZ1 expression was detected indirectly using anti-RIZ1 monoclonal antibody (1:25 dilution; Abgent, San Diego, CA, USA) and a FITC-conjugated secondary antibody following fixation and permeabilization with IntraPrep reagent (Beckman Coulter).

### Immunostaining

Immunohistochemical analysis of B5 fixed/paraffin embedded and decalcified bone marrow trephine biopsies and B5 fixed/paraffin embedded bone marrow aspirate clot samples was performed using an anti-RIZ1 monoclonal antibody (Abgent, San Diego, CA, USA) (1:25 dilution) and a horseradish peroxidase-coupled secondary antibody. RIZ1 expression in unmatched patient bone marrow biopsies and clot sections was calculated by measuring intensity levels of 3,3-diaminobenzidine chromogen staining (brown pixel intensity) that was normalized to the area scanned using an ACIS^® ^III scanner (Dako, Carpinteria, CA, USA). Statistical differences between chronic phase, accelerated phase, and blast crisis were determined using an unpaired *t*-test.

### RT-PCR

Total RNA was isolated from cell lines using the TRI-zol reagent (Life Technologies). cDNA was synthesized from total RNA using iScript cDNA synthesis kit (Bio-Rad Laboratories, Hercules, CA). cDNA was amplified in a 50 μl reaction containing Hotstar Taq DNA polymerase and buffer (Qiagen), 100 pmol primers (RIZ1: 5'-AACATGTGCTGCGAGGACTT-3' and 5'-TTCTTCCCTTTCCGGCTCT T-3'; β-Actin: 5' CCAAGGCCAACCGCGAGAAGAT-3' and 5'-TTGCTCGAAGTC CAGGGCGA-3'), and 0.25 μg cDNA.

### Statistical Analysis

All the data are reported as mean± s.d. The differences between the mean values were tested for statistical significance by the two-tailed Student's t-test (P-values).

## Competing interests

The authors declare that they have no competing interests.

## Authors' contributions

AL, NT and EP performed cell line experiments. ET performed immunohistochemistry. HMA and GG-M prepared CML tissue array, MC prepared matched CML patient material. JD and CRG designed experiments and wrote manuscript. All authors read and approved manuscript.
